# Roles of *Streptococcus mutans* in human health: beyond dental caries

**DOI:** 10.3389/fmicb.2024.1503657

**Published:** 2024-12-19

**Authors:** Yanke Fang, Xin Chen, Chun Hung Chu, Ollie Yiru Yu, Jinzhi He, Mingyun Li

**Affiliations:** ^1^State Key Laboratory of Oral Diseases, National Clinical Research Center for Oral Diseases, West China School of Stomatology, Sichuan University, Chengdu, China; ^2^Faculty of Dentistry, The University of Hong Kong, Hong Kong, Hong Kong SAR, China; ^3^Department of Operative Dentistry and Endodontics, West China School of Stomatology, Sichuan University, Chengdu, China

**Keywords:** dental caries, *Streptococcus mutans*, pathogenic factors, proinflammatory mechanisms, systemic diseases

## Abstract

*Streptococcus mutans* (*S. mutans*) is the main pathogenic bacterium causing dental caries, and the modes in which its traits, such as acid production, acid tolerance, and adhesion that contribute to the dental caries process, has been clarified. However, a growing number of animal experiments and clinical revelations signify that these traits of *S. mutans* are not restricted to the detriment of dental tissues. These traits can assist *S. mutans* in evading the immune system within body fluids; they empower *S. mutans* to adhere not merely to the surface of teeth but also to other tissues such as vascular endothelium; they can additionally trigger inflammatory reactions and inflict damage on various organs, thereby leading to the occurrence of systemic diseases. These traits mostly originate from some correlative findings, lacking a comprehensive evaluation of the impact of *S. mutans* on systemic diseases. Therefore, this review mainly centers on the dissemination route of *S. mutans*: “Entering the blood circulation - Occurrence of tissue adhesion - Extensive possible proinflammatory mechanisms - Concentration in individual organs” and analyses the specific effects and possible mechanisms of *S. mutans* in systemic diseases such as cerebral hemorrhage, inflammatory bowel disease, tumors, and infective endocarditis that have been identified hitherto.

## 1 Introduction

*Streptococcus* is the most abundant genus in the mouth, and as a member of this genus, *Streptococcus mutans* (*S. mutans*) plays a central role in the development of caries. It can synthesize extracellular polysaccharides through glucose transferase and promote bacterial adhesion and aggregation, leading to the formation of plaque biofilm and colonization in the oral cavity. Subsequently, *S. mutans* can metabolize carbohydrates to produce acids that act on dental tissues to demineralize and form cavities. The glucose transferase of *S. mutans* is highly compatible with the ideal adherent surfaces and sufficient carbohydrates in the oral cavity, thus playing a unique advantage.

However, when there are adherent surfaces and suitable carbohydrates in other parts of the body, the glucose transferase of *S. mutans* may still be functioning, and its other biological structures are also likely to be so. Furthermore, many studies have found that *S. mutans* can indeed enter the bloodstream with other oral microorganisms during dental extraction, root canal treatment, or even during daily toothbrushing (Li et al., 2000; Sonbol et al., 2009). Depending on the special biological structure, some special types of *S. mutans* that enter the circulation may not be recognized and eliminated by the immune system, but adhere to various sites of the circulation pathway, leading to inflammatory responses and tissue damage. In such cases, clinical practice may consider *S. mutans* as an important therapeutic target. Therefore, this review focuses on some of the pathogenic biological structures, possible pro-inflammatory mechanisms, and the complex and important pathogenic process, mechanisms, and factors of *S. mutans*-induced systemic diseases, with the hope of enabling clinical practice and fundamental research to have a more holistic understanding and recognition of the role of *S. mutans* in systemic diseases.

## 2 Pathogenic factors of *S. mutans*

*Streptococcus mutans* has many different surface biological structures that play a major role in the pathogenesis of oral and systemic diseases. For example, Rhamnose glucose polymer (RGP) is responsible for different serotypes of *S. mutans* (Yamashita et al., 1999), while glucosyltransferase, protein antigens, and polysaccharide-binding protease are the main surface protein antigens of *S. mutans*, which are essential for certain steps in the pathogenic process (Fujita et al., 2007). Additionally, *S. mutans* has many adhesins, and in the oral cavity, the adhesion of *S. mutans* is a key factor in the development of caries (Li et al., 2021), while in systemic diseases, adhesins represent an important advantage for host colonization.

### 2.1 Rhamnose glucose polymer and glucose side chain

*Streptococcus mutans* is classified into four serotypes (c, e, f, and k) based on the composition and structure of the cell wall-associated rhamnose polysaccharides. Serotype c is predominant in the oral cavity, with a detection rate of over 70% in the mouths of healthy subjects; serotype e constitutes approximately 20%, and serotype k constitutes less than 5% (Nakano et al., 2004; Nakano et al., 2007c).

In systemic diseases, RGP contributes to the resistance of *S. mutans* against phagocytosis by human polymorphonuclear leukocytes (Tsuda et al., 2000), and is capable of binding to fibronectin, type I collagen, and laminin. This enables it to aggregate at the endocardium, thereby enhancing the virulence of infectious endocarditis (IE) strains. In the IE rat model, the virulence level of the strain containing intact RGP is significantly higher than that of the RGP- mutant, and RGP can trigger platelet aggregation, which may result in vascular stenosis at the site of *S. mutans* aggregation (Nagata et al., 2006). Additionally, the glucose side chains also exert varying degrees of influence on pathogenicity. For instance, the serotype k strain of *Streptococcus* with a defective glucose side chain has been demonstrated to be less prone to phagocytosis by polymorphonuclear leukocytes, leading to prolonged bacteremia.

### 2.2 Glucosyltransferases (GTFs) and glucan-binding proteases (GBPs)

Glucosyltransferases have been implicated in sucrose-dependent adhesion of *S. mutans* to tooth surfaces. *S. mutans* strains produce up to three glycosyltransferases, Gtf-B, -C, and -D. From infected heart valve tissue, *S. mutans* strains lacking expression of each of the three GTFs could be isolated (Munro and Macrina, 1993; Nomura et al., 2006). Mice infected with isogenic mutant strains lacking all three GTFs had significantly reduced survival (Shun et al., 2005). Nucleotides with high homology to erythromycin and macromycin resistance genes were found in the *gtfB* and *gtfD* genes respectively. The insertion of these resistance genes resulted in loss of GTFs expression and acquisition of bacterial resistance, possibly contributing to the high virulence of the strains against cardiovascular diseases (Nemoto et al., 2008).

GBPs, owing to their glucan-binding attributes, constitute significant virulence factors in dental caries, jointly functioning with GTFs to facilitate caries. However, within the bloodstream, only a deficiency in GBPs has been discovered to extend the duration of *S. mutans* bacteremia (Nomura et al., 2004).

### 2.3 Protein antigens (PA)

In the oral cavity, this structurally complex, multifunctional adhesin mediates bacterial attachment to the salivary membrane of teeth through interactions with host scavenger receptors glycoprotein gp340 or protein DMBT-1 (Brady et al., 2010). In a rat bacteremia model, PA- *S. mutants* showed the strongest resistance to phagocytosis, exhibited stronger cell hydrophobicity, significantly prolonged the duration of bacteremia, and had significantly higher inflammatory markers than rats infected with the parental strain (Nakano et al., 2006b; Nakano et al., 2008; Matsumoto-Nakano et al., 2009).

### 2.4 Collagen-binding proteins (CBPs)

Collagen-binding proteins primarily consists of Cnm and Cbm, and CBP + *S. mutans* is more commonly isolated from dental plaque of patients with bacteremia and infective endocarditis. Approximately 15% of *S. mutans* isolates contain Cnm, while Cbm is rarely detected (Abranches et al., 2011; Nomura et al., 2012). The genes encoding CBPs are not uniformly distributed among serotypes and are mainly concentrated in strains e, f, and k (Lapirattanakul et al., 2011).

Cnm is a 120 kDa protein encoded by the *cnm* gene (Sato et al., 2004), which is found in 10–20% of oral bacterial strains and is most commonly found in serotypes f and k (Nakano et al., 2007a; Nomura et al., 2009). Because Cnm can also bind to type I collagen and laminin, it may produce similar effects to RGP. Therefore, Cnm is highly associated with bacterial attachment to blood vessels, and studies have shown that Cnm is a necessary condition for *S. mutans* to invade endothelial cells and subsequently cause cardiovascular disease.

Cbm is another CBP that is encoded by the *cbm* gene. Its hypothesized collagen-binding domain has 80% identity and 90% similarity with the homologous region of Cnm. Cbm is found in less than 3% of oral strains, most commonly in serotype k, where most lack PA expression (Nomura et al., 2012; Nomura et al., 2013). The type I collagen binding level of Cbm is higher than that of Cnm and can bind fibrinogen. Nomura et al. (2014) found that in the presence of fibrinogen, Cbm + /PA- strains had higher platelet aggregation induction and fibrinogen binding activity than Cbm- mutants. In the absence of fibrinogen, the platelet aggregation induction activity of Cbm + strains was relatively weak, as most CBP + *S. mutans* cells have a negative charge on their surface, which inhibits the aggregation of similarly charged platelets (Nakano et al., 2011). Therefore, for Cbm + mutant *Streptococcus*, the presence of fibrinogen is essential, and it may act as a bridge molecule to allow Cbm + mutant *Streptococcus* to bind to platelets.

### 2.5 Fibronectin-binding proteins

Fibronectin-binding proteins are cell envelope-associated proteins of *S. mutans*, including AtlA, RgpG, BrpA, and Psr (Lemos et al., 2019). Fibronectin-binding proteins mediate specific adhesion of *S. mutans* to fibronectin and endothelial cells (Chia et al., 2000). For instance, BrpA- *S. mutants* were more resistant to polymorphonuclear leukocyte phagocytosis and aggregation of platelets than the parental strain. The duration of bacteremia induced by this defective strain was longer in rats (Nakano et al., 2005).

The recently identified fibronectin-binding protein AtlA plays a role in *S. mutans* resistance to phagocytosis and survival in the bloodstream, and its deficiency results in reduced autolysin, prolonged chain length, and significantly reduced biofilm formation. Furthermore, physiological serum calcium concentrations promote its maturation. It is found to be associated with *S. mutans* survival and virulence to IE in a rat model (Jung et al., 2009).

## 3 Proinflammatory mechanisms

Inflammation is characterized by leukocyte migration from the vascular system to damaged tissue, regulated by proinflammatory cytokines. *S. mutans* infection can induce a series of inflammatory reactions, explaining systemic diseases caused by *S. mutans*. *S. mutans* surface PA has potential to stimulate TNF-α, IL-1β, and IL-6 production (Paik et al., 2003). TNF-α and IL-1β are proinflammatory mediators. And IL-6 leads to increased cell adhesion molecule and chemokine expression, attracting macrophages and neutrophils for microbial elimination as first-line defense.

### 3.1 Induction of TNF-α and IL-1β

In macrophages, *S. mutans* induces TNF-α and IL-1β production by activating signaling pathway ERK/p38/JNK and NF-κB through TLR2 (Toll-like receptor 2) and TLR4(Toll-like receptor 4), respectively (Kim et al., 2012). NF-êB activates a transcriptional response, leading to pro-IL-1β synthesis, and a second signaling pathway activates caspase-1-dependent IL-1β maturation and secretion via inflammasomes (Schroder and Tschopp, 2010).

TNF-α initiates a cytokine cascade and increases vascular permeability, recruiting macrophages and neutrophils to a site of infection. IL-1b is a cytokine produced mainly by activated mononuclear phagocytes, mediating host inflammatory responses in innate immunity. It has several biological effects, including phagocyte activation, antibody production, and T-cell polarization.

### 3.2 Induction of IL-6

IL-6 and its soluble receptors are key mediators regulating neutrophils and monocytes recruitment (Kaplanski et al., 2003). One study showed GTFs didn’t promote infectivity without glucan but affected inflammation. In acute inflammation, GTFs were the main regulatory protein inducing IL-6 (Chia et al., 2001). GTFs may stimulate IL-6 during systemic infection in the spleen and surrounding vegetations (Chia et al., 2002). Clinical investigation showed serum IL-6 levels significantly increased in all endocarditis stages, while other cytokines didn’t significantly increase (Rawczynska-Englert et al., 2000; Alter et al., 2002). Therefore, sustained IL-6 activation may promote myocardial injury. Additionally, as a gram + bacterium, *S. mutans* collagen combined with adhesive matrix molecules can inhibit the classical complement pathway (Kang et al., 2013).

## 4 Role in systemic diseases

### 4.1 Role in cardiovascular disease

*Streptococcus mutans* can cause damage to various organs and systems after entering the blood. At present, the mechanism of research on cardiovascular system damage is relatively clear. CBPs can bind with type I collagen to damage blood vessels, and cause cardiovascular diseases such as cerebral hemorrhage and infectious endocarditis, as well as inflammatory bowel disease, IgA nephropathy and tumor metastasis.

In various clinical and animal studies, *S. mutans* is frequently detected in heart valve and atherosclerotic plaque samples, and its role in causing bacteremia and vascular damage has been observed (Lockhart et al., 2004; Nakano et al., 2006a; Nakano et al., 2007b; Nakano et al., 2009; Oliveira et al., 2015; Nomura et al., 2020a).

Compared to healthy subjects, the detection rate of non-serotype c strains, such as serotype k, is significantly higher in the heart valves of patients with cardiovascular disease. Due to changes in cell surface antigens of serotype k strains, including high-frequency loss of PA expression and GbpA (Glucan-binding protease A) expression, as well as reduced GbpC expression, these strains can resist leukocyte phagocytosis and survive for extended periods in the bloodstream (Nakano et al., 2002). Importantly, serotype k strains highly express Cnm or Cbm, which are critical virulence factors for adhesion to vascular endothelial cells and heart valves, contributing to cardiovascular disease.

#### 4.1.1 Atherosclerosis (AS)

Atherosclerosis is a disease where lipid patchy deposits form in the walls of medium or large arteries, resulting in reduced or blocked blood flow. S mutans accelerated AS induction in mice through its damaging effects on vascular endothelial cells, which was more pronounced in the injured endothelium (Kesavalu et al., 2012).

It is now believed *S. mutans* infection of Human Aortic Endothelial Cells (HAECs) significantly upregulates intracellular TLR2 and NOD2 (nucleotide-binding oligomerization domain protein) expression, leading to greater cardiovascular disease-associated pro-inflammatory cytokine (IL-6, IL-8, MCP-1) production (Nagata and Oho, 2017). *In vitro* studies found upregulated cellular *DMBT1*(deleted in malignant brain tumor 1, proposed to be a tumor suppressor gene) expression in HAECs after *S. mutans* infection. *S. mutans* was more invasive to *DMBT1* knockdown HAECs, resulting in higher cytokine production. This suggests *DMBT1* inhibits bacterial adhesion to HAECs and provides protection against *S. mutans* infection (Oho and Nagata, 2019).

#### 4.1.2 Infective endocarditis

After *S. mutans* reaches the damaged cardiac endothelium, it binds to the exposed collagen, where activated platelets also bind via the factor vWF. When the Cbm + /PA- strain binds to the Glycoprotein IIb/IIIa receptor on platelets, it may interact with fibrinogen, accelerating the formation of redundant organisms, leading to the occurrence of IE (Koga et al., 1990).

Recently, Nomura et al. (2020b) suggested the type IV collagen-Cnm-*ARHGEF 38* pathway plays an important role in IE pathogenesis, explaining IE occurrence without heart valve injury. Activation and inactivation of small G proteins (A class of signal transduction proteins featuring guanylate binding sites and GTPase activity, and having a molecular weight of merely 20–30 kDa) involve cytoskeletal rearrangements in human cells (Cherfils and Zeghouf, 2013). Many bacteria invade human cells by expressing Rho guanine nucleotide exchange factors, activating small G proteins, and Rho GTPase-activating proteins (A protein involved in cellular signal transduction), inactivating small G proteins, to rearrange the cytoskeleton (Patel and Galán, 2006; Handa et al., 2007; Roppenser et al., 2009). Conversely, GTPase-activating protein may prevent Cnm + mutant streptococcal invasion by inhibiting type IV collagen binding.

#### 4.1.3 Cerebral hemorrhage

Eishi et al. (1995) showed in a mouse model that Cnm + *S. mutans* were also associated with worsening cerebral hemorrhage, a complication of IE occurring in about 10% of cases. A cross-sectional study showed Cnm + *S. mutans* increased cerebral microhemorrhages (CMBs) (Miyatani et al., 2015). A later clinical longitudinal study also showed Cnm + *S. mutans* associated with hypertensive cerebral hemorrhage and deep CMBs, two major hemorrhagic phenotypes of penetrating atherosclerosis (Hosoki et al., 2020).

Possible mechanisms of cerebral hemorrhage from Cnm + *S. mutans* are: blood-brain barrier permeability increases with age due to endothelial integrity decrease. Chronic hypertension also affects small vessel structure, leading not only to blood-brain barrier disruption, but also type I collagen accumulation. In addition, vascular changes from aging and hypertension release vascular factors attracting Cnm + *S. mutans* binding, promoting attachment to exposed collagen of small cerebral perforating arteries. Subsequent neutrophil infiltration and local inflammation exacerbation increase blood-brain barrier permeability and enzyme release (myeloperoxidase MPO-16 and matrix metalloproteinase MMP-9) accelerating endothelial damage (Nakano et al., 2011; Tonomura et al., 2016). Additionally, bacterial surface negative charge prevents infiltration of negatively charged platelets with collagen, promoting focal hemorrhage. Endothelial injury from aging and hypertension is more pronounced deeper, further progressing to cerebral hemorrhage and hemorrhagic stroke (Lammie, 2000).

### 4.2 IgA nephropathy (IgAN)

IgAN is characterized by predominant IgA1 deposition in the mesangial region, with extracellular matrix proteins composed of type IV collagen, fibronectin, and more (Kokubo et al., 1998; Narita and Gejyo, 2008; Abrahamson et al., 2013). In IgA nephropathy, the relationship between tonsil immunity and glomerulonephritis has been established. However, certain pathogens may impact tonsil immunity, resulting in IgAN (Komatsu et al., 2008). Mucosal changes like infection can activate the innate immune system, exacerbate existing IgAN, and promote disease manifestations like hematuria (Floege and Feehally, 2016). Higher type IV collagen concentrations could promote more pronounced aggregation of *S. mutans*. Some reports suggest that *S. mutans* can induce severe nephritis involving the glomeruli, tubules in rabbits, as well as IgAN-like glomerulonephritis in rats (Albini et al., 1985; Naka et al., 2021). Cnm + *S. mutans* strains are associated with elevated urine protein and severe IgAN, while the Cnm protein itself has no direct effect on the kidneys (Misaki et al., 2016; Ito et al., 2019). Immune reactions with IgA in Cnm + mucosal tissue may lead to glycosylation defects in serum IgA1 molecules, with glycosylation defense in IgA1 molecules as the primary pathogenesis of IgAN (Tomana et al., 1999; Rodrigues et al., 2017). This plays an important role in IgAN pathogenesis.

Hotta et al. (2001) proposed tonsillectomy and steroid pulse therapy for IgAN patients can not only reduce hematuria/proteinuria but also improve remission rates. This may support the significance of tonsillectomy in IgAN patient treatment and the importance of oral care in preventing kidney disease.

### 4.3 Non-alcoholic steatohepatitis (NASH)

”Two hit theory” is widely believed to be *the* mechanism for NASH development, where insulin resistance caused by metabolic syndrome from excessive nutrition leads to simple steatosis as the first hit. The second hit is oxidative stress, lipid peroxidation, and mitochondrial failure, accelerating liver inflammation and fibrosis, leading to NASH (Day and James, 1998). Compared to Cnm + /PA- variant *S. mutans* strain, Cnm + /PA + strain showed high virulence in aggravating NASH in mice (Naka et al., 2014; Naka et al., 2016). Cnm helps *S. mutans* adhere to liver cells without fatty acid accumulation. Cnm and PA may facilitate adhesion of *S. mutans* cells to hepatocytes without and with fatty acids, respectively. *S. mutans* expressing both PA and Cnm on the bacterial cell surface localizes in the liver and attaches to hepatocytes, leading to increased inflammatory cytokines related to oxidative stress, such as IFN-γ and metallothionein. This results in a second hit on liver cells, aggravating NASH (Naka et al., 2014). Increasing evidence supports clinical interaction between type IV collagen and Cnm + *S. mutans*.

### 4.4 Inflammatory bowel disease

Ulcerative colitis and Crohn’s disease are major inflammatory bowel diseases, which are chronic and recurrent intestinal diseases. Available data show that proinflammatory cytokines (TNF-α, IL-1β, and IL-6), and immunosuppressive IL-10 deficiency are markers of intestinal inflammation (Vasovic et al., 2016). IL-6 is the main cytokine in the inflammatory region of ulcerative colitis patients, and its concentration is related to the score of disease severity under endoscopy (Zdravkovic et al., 2014).

The aggravation of colitis may result from S mutans infection of the liver, IFN-γ released from the liver during S mutans infection is the first step for various inflammatory cascade reactions (Kojima et al., 2012; Kojima et al., 2014).

### 4.5 Tumor related mechanisms

**FIGURE 1:**
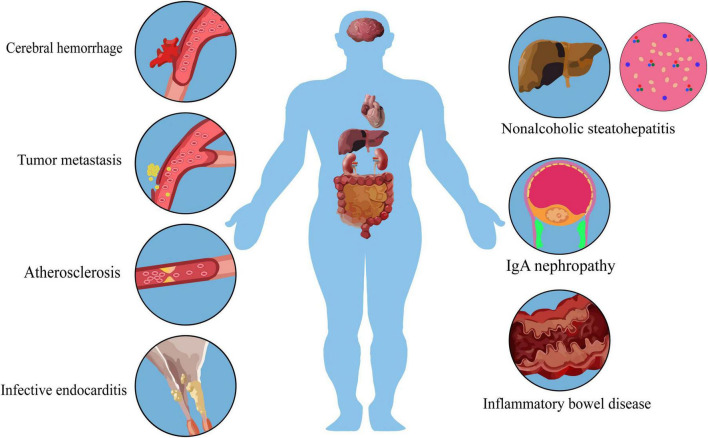
Multi-organ damage caused by *S. mutans* in systemic diseases.

*Streptococcus mutans* infection contributes to tumor invasiveness and is associated with poor disease control. In an oral squamous cell carcinoma mouse model, *S. mutans* promoted oral cancer development and progression by increasing IL-6 production (Tsai et al., 2022). *S. mutans* has the ability to induce vascular inflammation and disrupt the vascular barrier by upregulating IL-8, reducing vascular endothelial-cadherin expression, and enhancing intercellular adhesion molecule 1 signaling, thereby facilitating tumor cell extravasation and migration toward the endothelium (Yu et al., 2009; Yu et al., 2022; Figure 1).

The impairment of the vascular endothelium lead to cerebral hemorrhage, infective endocarditis and atherosclerosis. *S. mutans* can induce an upregulation of interleukin, which, in conjunction with its vascular damage, may contribute to the vascular metastasis of primary tumors.

During serotype k infection, the liver releases IFN-γ and triggers inflammation. This, combined with the interaction of type IV collagen, a basal membrane protein in parahepatic sinus cells, with *S. mutans*, can result in tissue fibrosis and non-alcoholic steatohepatitis. Simultaneously, IFN-γ released by the liver may be a primary factor in the exacerbation of colitis.

IgA immune reactions in Cnm + mucosal tissue may cause glycosylation defects in serum IgA1 molecules, with impaired glycosylation being the primary pathogenesis of IgAN. Immunofluorescence reveals IgA deposition in the mesangial region.

## 5 Conclusions and perspectives

The oral cavity is one of the most crucial interaction interfaces between the human body and the external environment. Oral microorganisms not only directly influence the disease status of dental caries and periodontal diseases but also exert crucial pathogenic effects in multiple organs. With the continuous advancement of metagenomics and high-throughput sequencing technologies, the relationship between oral microorganisms and systemic diseases has become increasingly explicit.

At present, frequent diseases caused by oral microorganisms in clinical practice include cardiovascular diseases, strokes, premature birth, diabetes, and pneumonia, among others. These diseases are frequently the outcomes of the concerted actions of several bacteria that inflict damage on the host. To precisely define the strict correlation between systemic diseases and oral microorganisms, it might be necessary to clarify the specific roles played by each type of oral microbe. *S. mutans* has its own distinctive features compared to other oral microorganisms, which enables it to cause not only the aforementioned diseases but also different systemic diseases such as IgAN and NASH.

However, precisely due to the unique features of each distinct species, the task of clarifying specific pathogenic mechanisms is colossal. In previous studies on *S. mutans*, many have remained at the stage of discovering correlations, lacking well-conducted clinical trials. Only a small portion, such as studies on cardiovascular diseases caused by *S. mutans*, have identified clear pathogenic mechanisms and perhaps intervention targets. On the whole, our understanding of the association between *S. mutans* and systemic diseases might still be in its initial stage.

In the future, it might be possible to carry out large-scale studies on diverse populations and carefully analyze the risk factors of different individuals, such as genetic background and lifestyle. Simultaneously, the future goal should not only focus on identifying more unidentified pathogenic mechanisms of *S. mutans* causing systemic diseases but also on the existing pathogenic factors of *S. mutans*. Measures to improve clinical treatment or other strategies should be adopted to address the adverse effects of *S. mutans*, such as maintaining good oral hygiene habits, undergoing regular dental examinations, and prophylactic use of antibiotics before treatment. A more comprehensive understanding and more rational responses to *S. mutans* will have an indelible significance for the prevention, diagnosis, and treatment of human systemic diseases.
